# Medical Opinion and Movement

**Published:** 1908-01-18

**Authors:** 


### Medical Opinion and Movement.

Some extensive researches have recently been carried out by Brugsch and Schittenhelm to determine the purin metabolism in gout. They have determined the purin contents of the blood of gouty individuals upon purin-free food, estimated the uricolytic ferment in the blood, observed the endogenous and exogenous uric acid and purin base outputs in chronic gout, examined the various tissues for uric acid, and carried out feeding experiments with glycocoll and alaran. They find that the endogenous uric acid content of the blood is raised in gout, and that the uric acid excretion is diminished. The metabolism of exogenous purins is altogether disturbed, the uric acid output being diminished and the purin basis relatively increased. As a result of their researches they are led to the conclusion that the gouty condition is due to a disturbance of the whole purin metabolism, causing a decreased uric acid formation, combined with a decreased uric acid decomposition. Uricæmia, therefore, constitutes an essential factor in the gouty diathesis, and it is as necessary to determine and control the purin metabolism in gout as the carbohydrate metabolism in diabetes.

The report of the Royal Commission on Tuberculosis has afforded such definite proof of the contamination of milk as a source of disease, and the question is of such vital importance in regard to the health of the children of the country, that no apology is needed for again referring to the subject in these columns. From careful investigations carried out by Dr. John McCaw, of the Belfast Hospital for Sick Children, the astounding facts are revealed that of all the children admitted into the chief children's hospitals of the United Kingdom during the year 1906, from 20 to 30 per cent. suffered from some form of tubercular disease. Further analysis shows that by far the greatest proportion of these patients suffered from *surgical* tuberculosis—that is to say, disease of the glands and joints, a form which has been definitely identified with absorption of the bovine bacillus through the intestinal canal. Moreover, a large proportion of these cases occur at an early age when milk constitutes the chief article of diet. The evidence, therefore, in favour of milk affording a serious source of infection with the tubercle bacillus for children is overwhelming. It is no longer a question of supposition. The evidence is clear and definite, and the facts are proved up to the hilt. It is, therefore, the duty of every public man and of every medical man to make an effort to put an end to this terrible and unnecessary sacrifice of child-life.

It has been publicly and authoritatively stated by Professor MacFadyean that as many as 30 per cent. of cattle in English cow-houses are diseased. Dr. McCaw estimates from this that about 6,250,000 quarts of milk is the daily yield of tuberculous cows in the United Kingdom! The amount of possible human tuberculosis from such a quantity of contaminated milk is appalling to contemplate. The only proper and obvious way to combat successfully such a condition of things is to institute a system of inspection and supervision of the dairies and farms of the country so as to control thoroughly the milk supply and secure the removal of all tuberculous cattle. We understand that a Bill is contemplated for the coming session with this object in view, but how far it will fulfil its purpose it is impossible at present to predict. In the meantime it rests with the profession to insist on the necessity of a thorough sterilisation of all milk consumed by young children. It is to be feared that in recent years there has been somewhat of a reaction against the use of sterilised milk. It has been clearly shown that milk when sterilised loses in nutritive qualities, is not so easily digested, and may even give rise to rickets. But under present circumstances there seems no alternative but to choose the lesser of the evils. Digestive troubles must be overcome. Even rickets is much less formidable than tuberculosis, and can generally be avoided by the addition of other ingredients, such as beef-juice, in the diet. At all costs, with our present contaminated milk supply, insistence cannot be too strong on thorough sterilisation.

An interesting medico-legal question suggests itself in relation to the Workmen's Compensation Act, namely, whether a workman can be compelled to undergo an operation, and whether, in the event of refusal, compensation should be reduced or refused altogether. In a recent case, at the Bangor County Court, it was decided to reduce the weekly compensation to a workman from 10s. to 2s., on the evidence that by the man undergoing a course of treatment, which hardly deserved the name of an operation, the man's condition might be so improved that he could earn much more than 10s. a week at the quarry. It has been already decided in the Court of Appeal that a man cannot be compelled, by suspending payment of compensation, to undergo an operation that is attended with a certain amount of risk. In Scotland, however, compensation to a workman has been reduced to a nominal sum on his refusing to undergo an operation which was alleged to be not attended with serious risk or pain. It would appear, therefore, that the view held in the law courts is that a workman must be prepared to undergo proper medical treatment within certain limits, but that he cannot be compelled to submit to an operation or other form of treatment involving an element of risk. It is obvious that in such cases there is rather a wide margin for individual judgment and opinion. Each case must, of course, be decided on its merits. It might be claimed that any operation involving the administration of an anæsthetic is attended with an element of risk, but this would, of course, vary according to the age and physical condition of the individual. Apart from this, there is the risk in regard to the result of the operation itself. A surgeon cannot always definitely promise the result he hopes to attain. When left to the choice of the workman, he will probably generally elect to "leave things to Nature."

